# Fear of Missing Out: Constrained Trial of Blockchain in Supply Chain

**DOI:** 10.3390/s24030986

**Published:** 2024-02-02

**Authors:** Roland Kromes, Tianyu Li, Maxime Bouillon, Talha Enes Güler, Victor van der Hulst, Zekeriya Erkin

**Affiliations:** 1 Department of Intelligent Systems, Delft University of Technology, P.O. Box 5031, 2600 GA Delft, The Netherlands; tianyu.li@tudelft.nl; 2 Supply Chain Finance Lectoraat, Business Media & Law, Windesheim University of Applied Sciences, Campus 2, Postbus 10090, 8000 GB Zwolle, The Netherlands; mfy.bouillon@windesheim.nl (M.B.); v.van.der.hulst@windesheim.nl (V.v.d.H.); 3 College of Engineering, Koç University, Rumelifeneri Yolu Sariyer, Istanbul 34450, Turkey; tguler17@ku.edu.tr

**Keywords:** blockchain, DLT, supply chain, transparency, traceability, digital transformation

## Abstract

Blockchain’s potential to revolutionize supply chain and logistics with transparency and equitable stakeholder engagement is significant. However, challenges like scalability, privacy, and interoperability persist. This study explores the scarcity of real-world blockchain implementations in supply chain and logistics since we have not witnessed many real-world deployments of blockchain-based solutions in the field. Puzzled by this, we integrate technology, user experience, and operational efficiency to illuminate the complex landscape of blockchain integration. We present blockchain-based solutions in three use cases, comparing them with alternative designs and analyzing them in terms of technical, economic, and operational aspects. Insights from a tailored questionnaire of 50 questions addressed to practitioners and experts offer crucial perspectives on blockchain adoption. One of the key findings from our work shows that half of the companies interviewed agree that they will miss the potential for competitive advantage if they do not invest in blockchain technology, and 61% of the companies surveyed claimed that their customers ask for more transparency in supply chain-related transactions. However, only one-third of the companies were aware of the main features of blockchain technology, which shows a lack of knowledge among the companies that may lead to a weaker blockchain adaption in supply chain use cases. Our readers should note that our study is specifically contextualized in a Netherlands-funded national project. We hope that researchers as well as stakeholders in supply chain and logistics can benefit from the insights of our work.

## 1. Introduction

According to [1], a supply chain can be defined as a group of individuals (such as an organization) involved in the upstream and downstream flows of a given product, service, finance, and information from a supplier to a customer. Today’s supply chains are considered highly complex networks that assist in transforming a raw material into a final product purchased by a customer. In 2020, the global supply chain management market was valued at USD 15.85 billion and estimates show that the market may double until 2026 [2]. The market impact of supply chain management is relatively high and its impact also has a significant effect on the daily lives of consumers, such as product disruption. While supply chain disruption is one of the most challenging issues in supply chain management, it also faces critical challenges such as customer demand for cost and faster response time, supply chain synchronization [3], and improvement of the traceability of flows [4].

Digitalization is an important concept for improving efficiency and increasing revenues. However, most supply chain use cases are based on traditional centralized entities: cloud services and server-based back-ends. More interestingly, we still see paper-based documentation and information sharing. For example, cable phone calls are also common for data exchange, according to [5]. Paper documents are fragile, easily lost, and difficult to store over the long term. Instead, a digitized supply chain can benefit logistics optimization, data availability, efficient management, etc. [6]. In addition, each organization participating in a supply chain has its own services. In many cases, the entities are connected in a hierarchical relationship, which imbalances the decision-making power of the entities. This imbalance creates unfair conditions for the economy to bloom further. This imbalanced architecture is due to the lack of transparency in information sharing between the stakeholders according to common agreements. In traditional systems, the majority of stakeholders cannot necessarily change the rules pre-described by monopolies.

One of the issues related to the supply chain is product flow traceability. As seen in numerous cases, the lack of reliable information about products throughout their entire life cycle causes not only economic damage but also health risks [7]. Unfortunately, traceability in today’s supply chains cannot be achieved in an immutable manner, which can lead to unexpected fraud in product delivery, costs, and stock. Moreover, the verification of the correctness of the data flow seems to be meaningless if the data traceability can be easily altered. The lack of immutability can also prevent digitization and the use of digital assets, which could instead bring significant economic growth to supply chains.

With the introduction of blockchain in 2008, immutable data structures attracted a lot of attention from the industry and academia. Stakeholders involved in supply chain and logistics are particularly interested in this technology as the supply chain involves multiple stakeholders who might benefit from sharing numerous types of data for better planning and prediction about their operations [8,9]. Blockchain initially offered a transparent platform with equal ownership, creating multiple opportunities for the stakeholders: data sharing, optimizing services, creating new services, and so on. Many were convinced that blockchain could democratize processes by providing equal ownership and transparency.

In a decentralized supply chain based on blockchain, it is believed that the involved stakeholders could have the same decision-making power (In traditional centralized systems, stakeholders’ decision-making power is not necessarily equal, since the consensus mechanism for decision-making is not processed according to a mathematically proven and secure operation, and stakeholders’ actions are not stored immutably. On the contrary, blockchain technology provides a secure consensus operation that can provide equal decision-making in terms of mathematical accuracy and security aspects, but it cannot eliminate possible collusion by stakeholders to influence decision-making). Moreover, supply chain-related data flows could be stored digitally and in an unalterable way, which could significantly increase the traceability of the flows and decrease the risk of fraud. Furthermore, the digitization of data sharing between entities could be a new source of income for supply chain members. Hence, there have been multiple European Union (EU) and national projects around blockchain, as well as proof-of-concepts developed for many use cases [10,11,12].

One such national project is the Spark! Living Lab project [12], funded by the Dutch Research Council, which aims to bring academic researchers and logistic companies together to exchange ideas and test their solutions around blockchain. When it was launched in late 2019, the project identified several use cases with the help of transportation companies such as conditional goods, the bill of lading, and creating a network of farmers who want to publish certificates for food production. These and many other use cases have been developed and proof-of-concept implementations were produced. Among the many use cases, only one proof-of-concept was managed to come to the testing phase in practice. Considering that the living lab was established to test many ideas around blockchain, this result was unexpected. Initially, the reason for the low number of tests, in practice, was associated with COVID-19: companies had serious problems and did not have the resources to test new and innovative solutions. However, critics from the scientific community on the use of blockchain were serious: many researchers suggested that there were better approaches than using blockchain in supply chain and logistics. What is more, a very important initiative between IBM and Maersk ended abruptly [13].

Given the number of academic publications on blockchain-based supply chain solutions, and numerous national, and international projects, it is curious that there is very limited deployment of the blockchain in supply chain and logistics that we see in practice. Obviously, one might assume that there are technical barriers to deploying blockchain technology to solve problems in supply chain and logistics; data validity is still not ensured, and data transparency is counter-productive for business. However, while there are proof-of-concept implementations for certain use cases, the argument of technical challenges only fails, at least for those use cases. Moreover, proposed solutions in the literature using blockchain are also not convincing.

In this paper, we want to find some answers to the question of why we have not seen blockchain-based solutions for supply chain and logistics while there have been multiple attempts in recent years. First, we explain how blockchain works and for what reasons it should be used. Second, we provide three representative use cases and analyze them in terms of technical, economic, and organizational aspects to understand whether they are suitable for developing a blockchain-based solution. Third, to better understand why we have not yet seen more deployment of blockchain in the supply chain field and to discover the reasons behind it, we present the results of a questionnaire answered by 18 people from companies in the field of supply chain and logistics. The questionnaire includes 50 different questions, as shown in Appendix A. Most of the questions are multiple-choice. Many of the questions were statements that companies had to agree with on a scale of 0 to 10. An important example of statement-type questions was: *If my company does not implement blockchain technologies, we will miss out on a potential competitive advantage*. It is worth noting that small, medium, and large companies were surveyed and half of them do not consider themselves blockchain experts. Finally, we discuss several key insights based on our experience over the years on blockchain-based research and the questionnaire result. We hope that the message in this work will help not only researchers but also decision-makers to make clear decisions for better investing resources for blockchain research and development.

The organization of this paper is as follows: In Section 2, we provide an evolving background about blockchain technology and its main characteristics. Furthermore, in this section, we give a high-level description of blockchain-based supply chains and logistics found in the literature and in industrial use cases already deployed. In Section 3, we describe three supply chain use cases and also highlight the main problems that traditional implementations of these use cases face. Furthermore, we show how a decentralized implementation can solve these issues. In Section 4, we provide an extended analysis of the responses to the questionnaire to highlight the companies’ state of blockchain technology in supply chain logistics. We discuss our study in Section 5, where we also highlight why we have not yet seen a blockchain-based solution in the supply chain logistics area, even though many believe there is significant potential. Finally, we conclude our work in Section 6.

## 2. Blockchain and Its Envisioned Use in Supply Chain and Logistics

In recent years, blockchain has been deemed as a potentially disruptive technology for supply chains [14], and an increasing number of research papers focus on applying blockchain to supply chains. Meanwhile, different works review and discuss existing solutions of blockchain for supply chains. In the following, we provide a high-level introduction to blockchain and highlight its use.

### 2.1. How Blockchain Works

Blockchain has been one of the most curious technologies among distributed ledger technologies in the last decade. As the name “distributed” implies, the ledger is distributed among all network participants, with each member holding the exact same ledger content and decision-making authority. As all members have the same content and, by default, everyone has access to the content of the ledger and the events that occur in the network, the technology ensures transparency.

All interactions with a blockchain can be performed through so-called transactions, which can contain different types of data. The transactions published to the blockchain must be digitally signed by the blockchain member’s private key. The sender’s public key is used to authenticate the transaction’s sender and ensures that the transaction has not been altered when sent. Thanks to the previously mentioned digital signature-based communication, the blockchain also ensures a certain level of authenticity.

When a member sends a transaction to the blockchain, the transaction’s validity is first verified by other members. After the validation process, valid transactions are collected and placed in a block, and then the new block is added to the blockchain according to a common agreement by the majority of members participating in that given agreement. This agreement is also called the consensus rule, and it is mainly based on complex mathematical algorithms and message exchanges between the participants of the consensus rule. For example, a block is considered valid if more than 2/3 of the participants agree on its validity. The most popular consensus rules are proof-of-work, proof-of-stake, practical Byzantine fault tolerance, and many others. For more information on consensus rules, we refer to the study by Bamakan et al. [15].

The most popular consensus rules can be categorized into proof-based consensus protocols, which include proof-of-work (PoW), proof-of-stake (PoS), proof-of-storage (PoStorage), and committee-based consensus protocols, which include the practical Byzantine fault tolerance (PBFT) [16]. On the one hand, in PoW, all nodes attempt to solve a complicated puzzle, and the node that first solves the puzzle will generate a new block. PoW was introduced by Nakamoto [17], and there is abundant literature on PoW-based consensus protocols considering scalability [18,19,20,21], decentralization [22], and security [22,23]. Compared to PoW, which gives the lead to the node with high computation power, PoS chooses the leader with a cryptographic random algorithm and the stakes that a node holds. PoS is more energy-saving compared to PoW, and there is also plenty of research on PoS [24,25,26,27]. Similarly, considering the storage of a node, there are also PoStorage-based solutions, such as [28,29]. On the other hand, committee-based consensus protocols (PBFT) achieve consensus through voting among the nodes. There is different research on both synchronous [30,31,32,33,34] and asynchronous [33,35,36] network models. For more information on consensus rules, we refer to [15,16].

In addition to the consensus rule, each block contains the hash value (i.e., a unique value, a digital fingerprint) of the previous block, which ensures the immutability of the blockchain, as changing the content of one of the previous blocks by a malicious actor, will result in a different hash value when adding a new block. Thus, the malicious behavior can be detected and the manipulated block will be rejected. Thanks to the blockchain immutability and the blockchain member’s authenticity, the non-repudiation feature is also ensured. In practice, it means that all the operations issued by a member are digitally signed, and the signature is infeasible to forge.

Bitcoin blockchain [17] was released in 2008, with the main objective of removing trusted third parties, for example, banks or third-party organizations from payment-related transactions carried out between two members. A trusted third party may behave maliciously by altering the payment amount, refusing to complete the transaction, or worse, overcharging the transaction fee. The trusted third party can be replaced by a blockchain, meaning that instead of trusting a single party, the user trusts that the majority of the members of the blockchain are motivated through its economic incentive.

Blockchain is a data structure that provides cryptographic guarantees for immutability, and by its construction, transparency, as identical copies of the data are stored by each node in the network. This data structure prompted V. Buterin to develop the Ethereum blockchain [37], giving birth to blockchain 2.0. The key novelty of this new era of blockchain lies in smart contracts [38], which are digital programs that can describe complete business logic and automate operations in a distributed way. Thanks to the trusted environment, the execution of smart contracts is safe, as the content of the smart contracts cannot be modified and their results cannot be altered.

Thanks to the trusted execution of smart contracts, the blockchain 2.0 era opened up a lot of opportunities for digital asset management and digital information monetization solutions. The execution of smart contracts is based on different platforms, such as Ethereum, Hyperledger Fabric, NEM, STELLAR, and Corda [39]. The application of different smart contracts across different platforms can utilize different aspects such as savings, security, confidence, and efficiency. Thus, we can see smart contract-based solutions for different domains, including healthcare, supply chain, energy, and more [39]. For more details about smart contracts, we refer the reader to the survey [39,40].

In the world of blockchain, two main categories of technologies can be distinguished: public ones, such as Ethereum or Bitcoin, and private ones (e.g., Hyperledger Fabric [41]). In the public blockchain, anyone can join and all members of the blockchain have access to read and write to the ledger. Most public blockchains require the presence of cryptocurrency in the blockchain, which is necessary because each modification of the ledger states involves a cost of *gas*, which is a certain amount of cryptocurrency used to reward the members participating in the execution of the consensus rule. It should also be noted that the public blockchains mainly contain a massive amount of participants and peer nodes hosting the ledger state. For example, Ethereum contains 8000 host nodes, also called peer nodes. Unlike public blockchains, private blockchains have a limited number of users, and new member registration in the network is allowed only under specific agreements, and in certain cases, only by a certificate authority. In addition, the number of peer nodes is also lower, as these blockchains are designed primarily for enterprise- and organizational-level use. Furthermore, these blockchains can apply read and write access to the ledger, and the presence of a cryptocurrency is not required as the transaction sending is usually free of charge. In general, the private blockchain provides a higher transaction validation/commit throughput than the public blockchains.

### 2.2. Blockchain in Supply Chain and Logistics

In 2020, Dutta et al. [42] reviewed 178 articles on blockchain in supply chain operations. The review mainly investigates different industrial areas, including transportation, finance, manufacturing, and energy. As a result, the authors illustrate the opportunities, challenges, and potentials in the combination of blockchain and supply chain. According to the review, the use of blockchain can benefit supply chains in terms of data management, transparency, response time, operational efficiency, disintermediation, immutability, and intellectual property management. With such benefits, blockchain can help supply chain functions such as provenance, resilience, re-engineering, security enhancement, business process management, and product management. Meanwhile, the authors summarize the challenges for the integration of blockchain with supply chains. On an organizational level, there is a lack of a common standard for different parties to share the same blockchain. On a technical level, there are issues with scalability, privacy, interoperability, product provenance, and latency. On an operational level, the system requires honest parties to participate without malicious actions. Also, trustful data storage, scalability, and adequate computing power are needed.

Reference [42] provides a detailed review of existing works with applications, challenges, and opportunities for the use of blockchain in supply chains. However, the paper notes that blockchain is always beneficial for supply chains. It lists around 80 real-world applications of blockchain in supply chains, but the number is limited considering the worldwide supply chain industries, and several are still being investigated. The authors only talk about the advantages of applying blockchain to supply chains without considering the drawbacks or whether blockchain-based solutions are superior to other solutions without blockchain.

On the other hand, Lim et al. [43] also emphasize that while there may be benefits of a blockchain-based supply chain, there are several ignored topics in the blockchain-based supply chain that need to be studied. The authors state that future research is needed to improve indicators of the social dimension in blockchain-based supply chains, such as working conditions, health and safety, human rights, and security. In addition to social impacts, more investigation is needed regarding sustainability management in blockchain-based supply chains. Similarly, Li et al. [44] show that data validation is usually not considered in blockchain-based supply chain applications. Nevertheless, their paper does not focus on whether blockchain suits different supply chain use cases.

According to [45], there is a significant lack of empirical research on blockchain-based supply chains, which is needed to highlight not only the information technology-related (IT) impacts but also the economics-related impacts in a blockchain-based supply chain. Furthermore, references [45,46] argue that with marketing theory, individual and organizational level analyses would also be useful in the supply chain research field.

Since 2018, many supply chain management-related works have been launched in industrial sectors. These projects were intended to apply blockchain technology to achieve secure and transparent shipping of goods and to use blockchain for information sharing and digital asset creation.

IBM and Maersk GTD Solution have launched the development of the TradeLens blockchain-based platform to enable more efficient and secure global trade in shipping goods. The main objective of the platform was to develop an open and neutral industry platform for the digitalization of the global supply chain. However, the project was discontinued in the first quarter of 2023 because the expected global industry collaboration was not achieved [13].

Automotive industry stakeholders such as BMW are also interested in investing in blockchain technology. BMW aims to create an open blockchain-based platform to enable secure and anonymous data sharing within supply chains. In addition, the PartChain project aims to ensure transparent and temper-proof data collection in the supply chain [47]. In 2022, the head of innovation and emerging technologies at BMW Group IT claimed that transforming the traditional system into a blockchain-based one might take longer than expected, but the technology still seems promising in supply chains [48].

## 3. Use Cases around Supply Chain

In this section, we focus on three use cases that have attracted significant attention from the industry related to the possibility of realizing distributed ledger technology during the initial project meetings in [12]. These use cases are (1) certificate dissemination for verified producers, (2) damage attribution of conditioned goods, and (3) electronic bill of landing. We provide a brief description for each use case, describe a design based on blockchain, and discuss the advantages and disadvantages of those designs. Please note that there are many other use cases in supply chain and logistics that can be considered for a more detailed study. The following use cases were the ones prioritized within [12].

### 3.1. Case 1: Certificate Dissemination for Verified Producers

Customers are increasingly more concerned with the origin of the products they buy as they become aware of ethical issues relating to the environment, sustainability, and forced labor [49]. To guide customers toward ethical consumption, certifiers have started auditing products (or their sellers), only awarding a certificate to products that satisfy predetermined requirements. Since certifiers are typically large centralized organizations, researchers have developed decentralized alternatives using blockchain technology to increase transparency in the certification process [50,51].

Certifiers audit entities such as sellers or their products. If the entity meets a set of requirements, it receives a certificate that convinces buyers of the level of quality. Certifiers exist because audits are resource-intensive and require expertise; it is not feasible for each buyer to perform an audit independently, nor is it wanted by the seller. Instead, buyers trust the certifier to perform audits on their behalf. Note that buyers usually judge the trustworthiness of a certifier based on external characteristics, such as its location, its source of income, its public reputation, whether it makes a profit, its size, how many certificates it issues, and its guidelines, among others.

For a certifier to be considered transparent, we identify the following set of required properties:

(1) **Authenticity:** A certificate for a given entity can only be issued and revoked by the certifier.
(2) **Revocability:** Buyers cannot be convinced by a certificate after it is revoked.
(3) **Transparency:** Buyers and sellers can find out when a certificate is issued or revoked.
(4) **Non-repudiation:** The certifier cannot deny issuing or revoking a given certificate.

#### 3.1.1. A Blockchain-Based Solution

We consider a use case in which a certifier provides certificates to potato farmers (sellers) who sell their goods to potato processors (buyers). The certifier, buyers, and sellers all take part in a permissioned blockchain, e.g., based on Hyperledger Fabric [41]. Buyers and sellers are enrolled by the certifier. The certificates are stored on the blockchain; proper user authentication is provided by the platform.

Obviously, any farmer can join this platform, and given that they provide proof to the certificate authorities, several certifiers can use this platform regardless of their geolocation. The system provides authenticity, revocability, transparency, and non-repudiation given that user authentication and revocability are integrated into the platform and the certificates are correct and sound.

#### 3.1.2. Alternative Solutions

Now, we want to investigate whether we can address the same challenge without using blockchain and propose a digital solution. A key point in this use case is that a local certificate authority (CA), or a certifier in our scenario, is essential in the process of providing certificates that acknowledge the claims by the farmers such as the quality of the soil, child labor, seed quality, and so on. For this certificate not to be forged, it should be digitally signed. Digital signatures provide *authenticity*, and they are massively scalable through the use of digital CAs. Many large CAs already provide *transparency* through the Certificate Transparency protocol. CAs ensure *revocability* by keeping updated lists that can be queried at any point in time by buyers [52]. Recent extensions of Certificate Transparency through the use of more complex verifiable data structures have been proposed [53]. These protocols are called Revocation Transparency and Certificate Transparency.

Thus, when a customer wants to check the quality of a product, it is sufficient to check the claims of that farmer by verifying the signature. As long as the signature is correct and revocation lists for certificates are up-to-date, this approach would work. Given that existing verifiable data structures are used, this setup satisfies all the conditions needed.

#### 3.1.3. Analysis

Now, we analyze the design using blockchain in three aspects: technical, economic, and operational.

**Technical aspect.** The main motivation for using blockchain is to provide a decentralized solution where ownership of the data stored on the blockchain is shared and each participant has equal rights in terms of adding new data. The platform is transparent and provides a democratic ecosystem where the involved farmers are not discriminated against.

Now, we want to argue that the key part of the blockchain-based solution is the local CA. Without CAs checking the facts in the physical world, the designed system does not work. Then, the question is, why are we proposing a blockchain-based solution? Does the blockchain improve farmers’ trust in the system? Our short answer is *No*. Here, the blockchain works as a data-sharing platform, solving the problem of easy access and revocation control, assuming that the revocations are also recorded on the blockchain.

Our assumption for using blockchain is that it provides equal ownership and transparency, as each party, in our case local CAs, has a copy of the data. However, this assumption does not hold since in the real world we have a network of local CAs; by definition, there is some level of trust toward the CAs. Assuming that CAs trust each other, there are alternative ways to share certificates: creating a hierarchical certificate authority structure as we use in real life or using cloud-based services that provide *verifiable logs* [53].

**Economical aspect.** Now, we will investigate the cost of using a blockchain-based solution. The initial idea is for the farmers to keep a local node at their premises. This means that the farmers install a workstation and have the means to run it with the proper security measures needed. There can be other solutions, such as having a single workstation for several farmers, but the challenge is the same: the upkeep of the workstation with proper security measures. Alternatively, we can assume that CAs manage a node in the network and a mobile application is provided to the farmers.

We ignore the cost of establishing a central authority to manage the certificates. Having a central entity is challenging to analyze in terms of the associated costs as there are many parameters to consider, such as the geographical locations of farms, the political situations in different provinces and countries, and the governance of these centers. We will leave this discussion to the experts.

**Operational aspect.** There are several existing systems to provide information on farm products [54]. Unifying all of these attempts—even a portion of them—is challenging in terms of business models and trust among different organizations. Clearly, the trust relies on the honesty of the certificate authorities.

Overall, with the mandatory involvement of CAs in the procedure, the blockchain becomes a data-sharing platform and loses its advantages. Then, the question is whether using the blockchain as a data-sharing platform offers any economic or operational advantage over existing approaches.

### 3.2. Case 2: Damage Attribution of Conditioned Goods

Transporting goods from the producer to the customer is the simplest description of a logistics chain. The corresponding payment then travels the route in the opposite direction. Within supply chains, many goods are stored and shipped under agreed conditions, usually with a service level agreement (SLA). This agreement guarantees under which conditions the payment takes place. Among many others, conditions such as the temperature and humidity during the shipment and the total time of the shipment are examples. In case some of these conditions are not met, a dispute needs to be resolved among the involved parties. With the development in sensory technology, it is believed that IoT devices and sensors can be deployed in containers, on packages, and even on individual products. Let us assume that sensory devices are available in the containers to measure temperature and humidity with location data and time stamps.

The condition of goods can be controlled in the following ways: continuously by an individual, continuously ambient, intermittently by an individual, or not at all. While the continuous individual monitoring of the condition of goods is the best approach for the highest oversight of goods, it is often impractical and expensive. Therefore, most supply chain managers opt for intermittent individual control of the condition of goods. For example, there can be two control points, one at the producer’s outbound dock and another one at the logistic service provider’s inbound dock. Those two points are considered representative, making it possible to identify deviations from the norm at those points, but not enough to identify deviations between the points. A solution for continuous control is definitely beneficial for multiple reasons. Using sensors, for instance, which are placed directly on the product, one can ensure continuous control.

#### 3.2.1. A Blockchain-Based Solution

In order to collect sensory data continuously and achieve this in a network of multiple stakeholders, each of which manages numerous transporters, a blockchain-based system can be designed. In essence, the sensors are connected to a blockchain governed by a coalition. The sensory data can be authenticated using digital signatures, and coalition partners can access the required data. We also assume that the sensory data are encrypted in such a way that only the relevant entities can access the data, as the information can be commercially valuable. This blockchain-based design is expected to convince the involved parties to have trust in the system, enabling different partners to collaborate for the transportation of goods. Such a solution can provide authenticity if digital signatures are used, and can be very beneficial for transportation companies in terms of the ease of governance since those products and containers with sensors are expected to travel across several borders.

#### 3.2.2. Alternative Solutions

One might argue that, in cases where we have IoT devices and sensors deployed in containers, on packages, and even on products, it is relatively easy to design a platform to share sensory data with the stakeholders. As we have existing trace and track applications for each transportation company, the existing platform can also report the conditions of the product delivery. Then, the question is whether we can convince the stakeholders to use a single platform or not. The best alternatives right now are either offline sensors inside the packages that track temperatures (not resistant to tampering) or stickers (damage indicators). For example, see [55].

#### 3.2.3. Analysis

**Technical aspect.** Using a blockchain-based solution or individual solutions relies on a single fact: the correctness of the sensory data. IoT devices and sensors are expected to be mounted to the containers, packages, or products, talking with the back-end via a mobile app of the driver or a reader. We also assume that the sensory data are digitally signed. However, the signatures can only provide the authenticity of the signer but not the correctness of the sensory data. Here, we *assume* the honesty of people who have direct access to physical IoT devices and sensors. Under this assumption, the digital solutions explained above can provide the required functionality. On the other hand, if this assumption is not correct and the sensory data can be manipulated, neither of the solutions can perform. The only approach we can think of to overcome such a situation is to deploy different sensors with countermeasures such as deploying multiple sensors, using a trusted execution environment (TEE) [56] and anomaly detection [57].

**Economical aspect.** At the moment, each transportation company has a track and trace system, and we assume that updating such systems with sensory data will require the deployment of sensors and connecting them with the existing digital systems. Deploying blockchain-based systems is not needed for a single company. We envision a case where multiple companies agree on such a system in case there is a significant financial gain, which is beyond our capacity to judge.

**Operational aspect.** Convinced by the economic or governance benefits, the above blockchain-based solution is developed for the Spark! project [12]. Unfortunately, the technological solution could not be tested in practice. The end-users of the solution identified two reasons for the failure of deployment: Firstly, the main supporting stakeholders of the idea shifted their focus to internal priorities due to COVID-19-related challenges from deploying emerging technology. Secondly, the involved partners were not convinced about the advantages of such a system, in terms of transparency and equal rights for governance. Even worse, the stakeholders were worried about potential financial loss. We will investigate this point further in Section 5.

### 3.3. Case 3: Electronic Bill of Ladings

The bill of lading (BoL) is a title document, a receipt for shipped goods, and a contract between a carrier and a shipper. This document is issued by a carrier and is passed on to the shipper when the goods are loaded. This process takes place physically. Considering the global international supply chain, information processing and the validation of the BoL are significantly challenging. Transactions often involve multiple parties who are mutually distrustful of each other, with goods traveling between multiple ports. Furthermore, the individual systems that each company uses cannot be easily made interoperable, as there are currently no common standards for data exchange.

The limitations of the current process have become glaringly visible in recent years, particularly during the COVID-19 crisis. The finance of international trade relies heavily on paperwork and manual processes as a way to provide proof of existence or ownership. Warehousing receipts, letter-of-credits, and BoL depend on courier services for the transfer between participants in a transaction. Finally, the transfer of the title and, in particular, the consequent use of the BoL as collateral in trade finance, is a strict *paper-only* process owing to statute law (e.g., the Dutch Civil Code) requirements in most countries. Not only is the current process inefficient, but it is also far from secure.

#### 3.3.1. Blockchain-Based Solution

TradeTrust, developed in [58], is a digital protocol with a predefined set of standards along with governance and legal frameworks. It works by separating the tracking of legal ownership from the content of the document. While the digital document and its underlying data are stored off-chain under the strict control of the original data owner, the ownership is securely managed on a distributed ledger. Users are free to decide how they want to digitally exchange the BoL data. Each document can be identified uniquely by a document hash. A hash acts like a fingerprint of a piece of data: every (unique) piece of information generates a unique hash; it is infeasible to obtain the original data back from only the hash. Furthermore, any electronic BoL (eBoL) can be serialized and is not restricted by data standards, ensuring integration with any existing data exchange formats. The system can verify ownership using a smart contract. According to TradeTrust’s Singularity Principle, as long as the token framework is properly implemented, it is practically infeasible for a hash (and, therefore, the reference to a document) to be duplicated. This is partially due to, e.g., Ethereum’s necessity for smart contracts to have unique addresses, and partially because the smart contract itself guarantees that a hash can only be registered once in the registry.

#### 3.3.2. Alternative Solutions

An ideal system would be an electronic bill of landing for global use. Such a system would require establishing a central authority that multiple countries trust. As our readers can easily see, this is a political challenge and not easy to solve. A partial system with several ports from different countries still has the same challenge: establishing a mutual trust to use a central system.

#### 3.3.3. Analysis

**Technical aspect.** TradeTrust showed that the creation of a sandbox environment (involving the first legally binding, interoperable, paperless transfer of ownership of an eBoL) can be realized technically. The first TradeTrust-enabled transfer of ownership of an eBoL was performed back in March 2020. With several parties involved, the project has now evolved with several solutions based on its foundations. Applications such as Quay Connect [58] are under active development, with transactions being made daily, introducing efficiency in terms of processing documents digitally compared to conventional solutions.

**Economical and Operational aspects.** TradeTrust provided exemplary success for a problem that had no existing solution. We believe the motivation behind this success can be explained as follows: There is a need to replace the current, inefficient, insecure system, and there are multiple stakeholders who need such a solution due to economic reasons: better logistics planning for port management and detecting criminal acts, e.g., transportation of illegal material, required by law. Clearly, a blockchain-based solution provides transparency among its users.

The blockchain deployment status in the previously elaborated use cases, the trust model of the system, the supply chain use case based on, and the outcomes related to the blockchain adoption are summarized in Table 1.

## 4. Evaluation

Blockchain was considered a disruptive technology, particularly for supply chain and logistics. Yet, the deployment of blockchain-based use cases is very limited. In order to investigate whether the reasons are related to technical challenges or some other reasons, we reached out to supply chain companies and asked several key people to fill out a questionnaire (see Appendix A), which was sent directly via e-mail to company contacts and published on social media. The questionnaire had 50 questions on 3 different aspects: (1) the background of the company and the people, (2) the background on digitalization and blockchain, and (3) the perception of blockchain for their business (challenges and opportunities). Now, we will provide the results.

### 4.1. Company and Interviewee Background

Table 2 summarizes the background information of the interviewed Western European companies due to the fact that the study was supported by a Dutch National Funding agency. For our questionnaire, we received input from 18 employees. The respondents belonged to 4 different age groups: 6 people were between 21 and 30 years old, 5 individuals were over 31 but under 40 years old, 3 people were between 41 and 50 years old, and finally 4 individuals were over 51 but under 60 years old.

Moreover, 7 out of 18 interviewees worked in managerial positions, while the rest of the group consisted of business analysts and specialists. Half of the interviewees confirmed that their level of blockchain expertise was greater than 5 on a scale of 10. Out of 18 companies, 7 were small-sized, 6 were medium-sized, and 5 were large-sized enterprises. Only half of the surveyed companies had conducted blockchain-related projects in the past. Four companies provided technology for supply chain and logistics companies.

### 4.2. Position on Digitalization and Blockchain

In terms of deploying technological innovations, 5 companies were active in creating and testing new ideas related to digitalization, 6 companies used basic digital technologies only for internal affairs, and 7 companies reported that data sharing with suppliers and clients is important. A striking 72% of the companies, i.e., 13 companies, reported that they could use data analytics to improve and transform their business. However, an essential question is on the use of data: *For what reason are the data used?* We received the answers, as presented in Table 3.

In terms of data sharing, the results are diverse, as summarized in Table 4: 50% of the companies share data with their customers, and only 6% use the data internally. According to the results, 22% of the companies still use paper, 38% use phones, and 89% use e-mails to share data. The majority of the companies, 55%, use cloud-based systems.

In our questionnaire, we also asked about the impact of a potential data leakage: More than 61% of the companies reported this as a serious risk.

The companies were also asked about their stakeholders participating in information-sharing operations. Table 5 highlights the percentages of companies (retrieved from a total of 18 companies) performing information-sharing operations with the stakeholders listed in the same table.

According to the respondents, the majority of the companies, 78%, use paper contracts to execute an agreement with other parties in the supply chain; 22% of companies also use verbal communication, and another 22% use smart contracts for this purpose. Moreover, 17% of the companies also execute agreements via third-party organizations.

The interviewees’ answers also highlight that 78% of the companies use a cloud-based system for online data storage, and nearly 64% also use an API (i.e., an application programming interface, usually a software interface to exchange information between separate applications or IT systems) and other methods, such as the enterprise resource system (ERP), warehouse management system (WMS), transport management system (TMS), and electronic data interchange (EDI). One-third of the companies also apply connected sensors and IoT for data collection, and only 11% of the companies utilize AI (artificial intelligence) and blockchain technology.

While only half of the respondents confirmed that they had already developed a project around blockchain, the rest of the respondents also had a perspective on what blockchain technology could offer to their business. Blockchain’s main features, including “Transparency for product or service flow”, “Traceable data”, and “Connecting all parties of the supply chain within the same network”, were known by more than two-thirds of the respondents. Other features, such as “Single source of truth” and “Secure business agreement via Smart contracts” were admitted, by two-thirds of the interviewees. The “Security”, “Payments” and “Immutable data” features were obvious to 61% of respondents, while only half of the interviewees were familiar with the “Decentralized verification”. Surprisingly, the features “Shared or equal data ownership” and “Inbuilt resilience and fault tolerance” were only known by less than 38% of the companies (i.e., only the blockchain experts noted these features).

As with every technology, blockchain technology also has its disadvantages, companies were asked about the disadvantages already known. Half of the companies knew about “Transparency ruins business cases" and more than half of them were familiar with the “No one size fits all (No generalization, case-specific solutions)”. Two important disadvantages were also identified by the companies: “Need for active participation” and “Lack of understanding and implementation”, 67% and 78% of the companies admitted these two drawbacks, respectively.

More than half of the surveyed companies agreed that the level of security of blockchain-based systems is higher compared to the current system. Nearly 39% of respondents do not know whether blockchain-based systems are more secure, and one company finds the blockchain-based system less secure than the current one.

### 4.3. Challenges and Opportunities of Using Blockchain for Supply Chain Logistics

More than 61% of companies surveyed see the implementation or replacement of existing systems with blockchain as a barrier to further investment in blockchain technology. Half of the companies interviewed report that the broad adoption of the technology is also slowing corporate investment in blockchain technology. In addition to the aforementioned barriers, the companies also say that another major issue is insufficient in-house knowledge or expertise.

Companies also shared information about the current relevance of blockchain to their businesses; 39% of companies believe that blockchain is important, 28% find it relevant, and the remaining respondents are unsure or have no idea (3 unsure and 3 companies have no idea, respectively).

Other important points were also highlighted in terms of company investments in blockchain technology. One-third of companies will invest in blockchain technology exploration in the near future. Two companies intend to invest in blockchain technology exploration in at least 2 years and three in at least 5–10 years. Finally, 22% of respondents have no idea about investing in technology exploration and 17% do not want to invest. To gain more insight into potential corporate investments in blockchain technology, we asked companies about the amount of annual revenue they might invest; 11 out of 18 companies can only spend 0.5% of their annual revenue on blockchain investment, and 2 can only spend between 0.6% and 1%. The remaining five companies can spend more than 5.1% of their annual turnover on this investment.

The companies were also asked about their actual or future participation in a blockchain consortium with other companies. Only 3 out of 18 companies have already participated in such a blockchain-based consortium and only 2 companies have considered joining a consortium; 7 companies reported that they were unsure and the remaining 6 companies reported no interest in participating.

A total of 70% noted that their companies are leading or on par with their competitors. However, the remaining 30% are not considering participating in blockchain efforts. Table 6 shows the state of adoption of blockchain by companies compared to their direct competitors.

**Figure 1:**
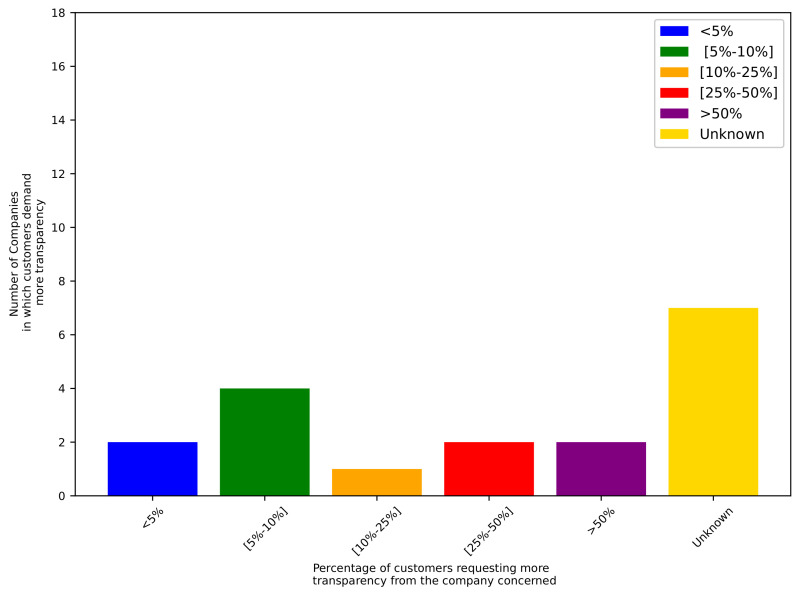
Percentages of customers requesting more transparency from the companies concerned.

With 11 companies, the customers have demanded increased transparency in the companies’ supply chains. The percentages of customers requesting enhanced transparency from the companies are represented in Figure 1 in a graph. The x-axis contains the different ranges of percentages of the customers requesting more transparency from the companies concerned. The ranges are as follows: less than 5%, between 5% and 10%, between 10% and 25%, between 25% and 50%, greater than 50%, and finally, an unknown range, indicating that the company has no information on the percentages. The y-axis represents the number of companies within the percentage ranges described above.

The scale of customers requiring transparency is various; only two companies claimed that more than 50% of the customers needed more transparency. In two companies, more than 25% but less than 50% of the customers had this need. Between 10% and 25% of customers wished to have more transparency in only one company among the interviewees. Four of the companies ensured that between 5% and 10% of their customers required more transparent operations to be declared. Finally, two companies specified that less than 5% of their customers needed more transparency. Surprisingly, the seven other companies were not able to provide any information on demands for enhanced transparency.

Regarding transparency objectives, it is critical to know which blockchain models companies intend to use. Therefore, we asked each company what blockchain model they used or will use. The answers are presented in Table 7.

Finally, the companies were also asked about their level of agreement or disagreement (5: strongly agree, 1 strongly disagree) on the following statements:

(a) New revenue sources from blockchain and/or digital asset solutions will be seen in our sector.
(b) If my company does not implement blockchain technologies, we will miss the potential for a competitive advantage.
(c) Blockchain and/or digital assets solutions or strategies are being discussed by our business partners, suppliers, customers, and/or competitors.
(d) There exists a compelling business for utilizing blockchain technology and/or digital assets in my organization.
(e) Blockchain technology is broadly scalable and has achieved mainstream adoption.
(f) Our executive team believes blockchain and/or digital assets are overhyped.
(g) Blockchain solutions are being discussed or developed by suppliers, customers, and/or competitors to address problems in the value chain.
(h) The industry will be disrupted by blockchain technology.

**Figure 2:**
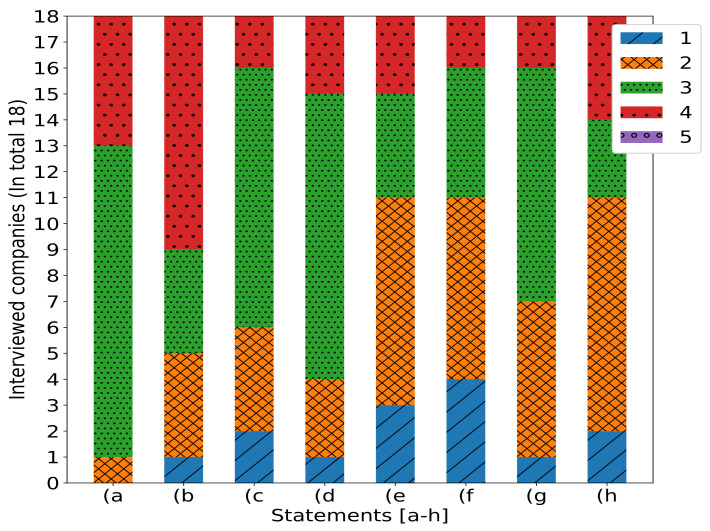
Companies’ opinion about the statements provided in the list on the previous page.

The answers to these statements are presented in Figure 2. The grouped bar chart displays the responses of 18 interviewed companies to the previously described statements (a–h). Each bar is categorized into five levels (1–5), indicating the degree of agreement or disagreement. Companies generally exhibit varied responses to statements (a–h).

## 5. Discussion

In this section, we address the puzzle surrounding the limited prevalence of blockchain-based solutions in the supply chain logistics sector, despite their widely recognized potential. Initially, we conducted a systematic analysis of questionnaire responses to discern technical, operational, and economic factors influencing deployment decisions. Subsequently, our focus shifted to the practical application of these insights within the use cases of the Spark! project, allowing for a grounded reassessment of our initial hypotheses.

In light of Figure 2, it is evident that there exists a level of uncertainty regarding the potential new revenue streams stemming from blockchain and digital assets. This ambiguity could conceivably impede the pace of blockchain adoption and associated investments.

The companies involved are grappling with uncertainty regarding the extent to which discussions on blockchain or digital asset solutions should be disclosed to business partners, suppliers, customers, or even competitors. This deliberation carries the potential risk of necessitating changes to established business models or partnerships, possibly leading to customers migrating to alternative providers. Moreover, the absence of a definitive and persuasive business case for implementing blockchain in this domain further complicates matters. We will delve into this issue in greater detail in subsequent sections.

What is intriguing is that, despite the involved companies not perceiving blockchain and/or digital assets as overhyped, they do not foresee a disruption of the industry by blockchain technology. A prevalent belief is that blockchain still lacks broad scalability and is yet to attain mainstream adoption. These factors collectively impede widespread adoption and amplify the associated investment risks.

The responses from the companies underscore several impediments to embracing blockchain technology, including reluctance to replace existing systems or implement a blockchain-based one. Additionally, the lack of in-house expertise and the limited endorsement of the technology by the board are identified as significant barriers to new investments. Interestingly, only 61% of the companies acknowledge that their clients are advocating for enhanced transparency, a benefit that can be realized through the adoption of blockchain technology.

The variance in the proportion of customers seeking enhanced transparency from their respective companies is notable. These results unveil two pivotal insights: Firstly, the fact that 7 out of 18 companies were unable to furnish any information suggests that these companies may not have thoroughly examined their customers’ present and future requirements. Conversely, the customers themselves may be unaware that blockchain applications can indeed facilitate enhanced transparency in supply chain and logistics. They might also harbor doubts about their chosen company’s proficiency in working with blockchain technology. Within the supply chain and logistics sphere, a multitude of stakeholders come into play, including producers, local communities, suppliers, companies, and customers. Enhanced transparency in this realm holds immense societal potential, offering deeper insights into the environmental context of production. This encompasses factors like community involvement, production sustainability, and pollution levels associated with transportation. Human–computer interaction research stands poised to empower customers to recognize the advantages of augmented transparency in supply chains and logistics. Related work [59] shows that information pertaining to supply chains influences customers’ product choices. The study further indicates a growing tendency among future clients toward environmentally conscious producers. Expect a rise in new customers advocating for increased transparency, thereby driving companies toward more transparency in their supply chains and logistics. Moreover, a holistic, multi-disciplinary approach, fostering robust collaboration between economics, human–computer interaction, and engineering disciplines, is essential. This approach will aid companies in discerning the utility of understanding their customers’ transparency needs, the reciprocal benefits of transparency, and the socioeconomic impacts achievable through expanded transparency of supply chain and logistic use cases realized by blockchain technology.

At the same time, many are still unsure if suppliers, customers, and/or competitors will make blockchain development address problems in the value chain. Finally, half of the companies agree on missing the potential for competitive advantages if they do not implement blockchain-based solutions in their businesses, demonstrating ***fear of missing out***. Since several blockchain characteristics, such as “Security”, “Payments”, “Immutable data”, “Decentralized verification”, and “Shared or equal data ownership”, are only known by less than two-thirds of the companies, this leads us to believe that the companies are not knowledgeable on why blockchain can be useful to their businesses.

The findings strongly indicate that companies lack a comprehensive understanding of the main advantages and drawbacks of blockchain technology. This knowledge gap may indeed impede the seamless integration of blockchain technology at the organizational and enterprise level. Moreover, a human–computer interaction study can shed light on the significant role of human decision-making in addressing the integration challenges posed by this technology. This suggests that an individual’s familiarity with the clear benefits and drawbacks of blockchain technology will not only hold economic ramifications, influencing the company’s overall success, but also carry societal implications. Given that every participant within the supply chain and logistics network is impacted by the decisions made by individuals within a company, it is imperative to recognize this connectivity. Furthermore, we posit that undisclosed advantages known only to the decision-maker have the potential to disrupt the overall integration process, with potentially negative repercussions for multiple stakeholders within the supply chain and logistics domain. Therefore, we advocate for companies to adopt a human–computer interaction-centered approach in training their employees on blockchain technology. We refer to a recent study [60], which proposes interactive workshops as an effective means to enhance understanding of blockchain technology.

Furthermore, according to the results, we also notice that the majority of information sharing takes place between the “Distributors”, “Transporters”, and “End Users”. In future studies, these three stakeholder groups could be targeted to find out more about their specific supply chain and logistics needs, e.g., more transparency, more secure data sharing, and immutable data. The study could help one to better understand whether these three stakeholders are more suitable for participation in a blockchain-based supply chain and logistics system compared to other stakeholders.

As a limitation of our study, the companies that responded to our questionnaire are all based in Western Europe. Future studies could help to highlight the situation of supply chain and logistics integration with blockchain technology in other countries. Furthermore, a study focusing on North American companies could provide more information on the future of blockchain in supply chain development, due to the different economic and investment strategies applied by these companies.

Considering real-world attempts at using blockchain, we see that the questionnaire results are highly correlated and provide clarity on the reasons why we do not see real-world deployment. For example, the decision by IBM and Maersk GTD Solutions to discontinue the development of their blockchain-based supply chain platform was due to the lack of global industry collaboration. Even though these two industry giants seem pessimistic about the development of blockchain in their use cases, it is probable that the transformation in other use cases will happen sooner or later, as the companies argued that they could lose their competitive advantage over a competitor if they did not invest in blockchain, and that blockchain and digital assets are not overrated. Furthermore, the head of innovation and emerging technologies at BMW Group IT stated that blockchain technology in the supply chain is a promising solution but the transformation of traditional systems into a blockchain-based one might take longer than expected [48].

Based on the insights gleaned from the questionnaire results, we will now scrutinize the three identified use cases. The first two use cases currently operate with functional systems. In order to use blockchain, there should be a clear motivation for stakeholders to deploy a blockchain-based solution, e.g., economic or legal aspects. Furthermore, even if the stakeholders want to deploy such a system, these two use cases heavily rely on trust in certificate authorities and sensors. In practice, the blockchain tends to exclusively serve as a data-sharing platform. It is in this context that we underscore the challenge of engendering a cyber-physical system. Establishing an immutable digital representation of a physical object constitutes a pivotal step in the development of digital systems, encompassing blockchain-based solutions. While this issue is not directly tied to blockchain technology, resolving this challenge could lead to a new era of digitalization across various domains, including the realm of blockchain-based solutions, like those found in circular economy initiatives [61]. In all the aforementioned assertions, we predicate our assessments on the assumption that the cyber-physical system stands as a trustworthy and reliable source of data.

For the third use case, however, there are several reasons to use blockchain: there is no existing system that is used by multiple stakeholders; there are legal and economic reasons to monitor containers at ports globally, as much as possible. Such a system desperately needs transparency and trust, which makes blockchain a suitable technology. However, even though cyber-physical challenges still exist for this use case, the incentive for stakeholders to use such a solution is sufficient enough to oversee that issue.

Our study holds significant implications for the MDPI Sensors readers; specifically, our findings contribute to the knowledge base in blockchain technology and its application and limits in the supply chain and logistics field. Throughout our research, we collected valuable insights that extend beyond the immediate scope of our study. These lessons are pertinent to professionals, researchers, and practitioners in the blockchain and supply chain field, and they include three pivotal conditions for the broader implementation of blockchain-based solutions in practical applications. *(1) The absence of existing solutions*: The presence of an established digital system can impede the adoption of blockchain-based solutions. Stakeholders often hesitate to incur the costs and risks associated with transitioning to a new approach, especially when it involves cooperation with competitors. *(2) Clear benefits of deployment*: Implementing blockchain-based solutions should offer clear advantages, including economic gains, legal compliance, and noteworthy social impacts on the actors within the supply chain. *(3) Resolution of cyber-physical connectivity*: This entails either placing trust in a real-world entity like Certificate Authorities (CAs) or devising a technical solution, an undertaking yet to be accomplished.

Our methodology is essentially based on the creation of the questionnaire used to survey companies involved in supply chain and logistics. The advantage of the questionnaire is that it contains 50 diverse questions, allowing it to highlight the different issues that might influence the adoption of blockchain technology in supply chain and logistics. As a limitation, the results of the questionnaire can certainly not reflect a global situation of blockchain adoption since the number of companies participating in the questionnaire is limited and they are located in specific areas. However, the slow adoption or complete halt of projects led by industrial giants (i.e., BMW, IBM, Maersk) shows a strong overlap with the results obtained by the questionnaire, which may also imply that our methodology could be valid to highlight the global situation of blockchain adoption in supply chain and logistics. Our cross-analysis includes an in-depth examination of the questionnaire responses, the current situation of blockchain adoption in supply chain projects led by major industrial companies, and the blockchain adoption opportunities in projects based on traditional implementations. The companies surveyed had an interest in responding because we promised that the results of the questionnaire would be published, implying that companies would receive meaningful feedback from future readers. We believe that feedback from the research community, those involved in supply chain and logistics, and other blockchain users will validate our results satisfactorily.

## 6. Conclusions

In this work, we sought to identify why we have not yet seen more deployment of blockchain-based solutions in supply chains and logistics. For this purpose, first, we studied three existing supply chain use cases and highlighted the benefits and disadvantages of adopting blockchain in these projects. Second, we designed a questionnaire with 50 questions and invited 18 companies from the supply chain field to respond. Our goal was to understand the views of practitioners and experts from the field about this issue.

According to the results of the questionnaire, the application of blockchain in supply chain use cases in the future appears to be bittersweet. On the one hand, in the future, we might see more blockchain deployment in supply chains, as half of the companies interviewed agree that they will miss out on a competitive advantage if they do not invest in blockchain technology. On the other hand, the companies also argue that blockchain technology has not yet been broadly deployed and has scalability issues. The results of the questionnaire also demonstrate that the companies’ judgments to invest in the technology might be due to limited knowledge of the real benefits and drawbacks of blockchain technology in supply chains. For example, only 38% of companies were familiar with the following blockchain features: “Shared or equal data ownership” and “Inbuilt resilience and fault tolerance”. Furthermore, the future integration of blockchain technology in supply chains is probably not hopeless, as customers of 61% of the companies are asking for more transparency, which can be realized in a more reliable and immutable manner thanks to blockchain technology. Overall, we see that there are technical barriers, as well as economic and operational concerns. Hence, we believe that blockchain still has potential in supply chain and logistics, given that all three aspects are satisfied.

With this work, we would also like to motivate supply chain and logistics companies to follow research studies to better understand what blockchain can provide as a benefit for their business growth, and more importantly, how a blockchain-enabled supply chain can influence human actors involved in supply chain and logistics, such as local communities, as well as which environmental impact can be achieved when the blockchain-based supply chain provides enhanced transparency. Therefore, we look forward to seeing more multi-disciplinary research in fields such as economics, engineering, and human–computer interactions to facilitate blockchain adoption in supply chain and logistics.

## Figures and Tables

**Table 1:** Blockchain deployment status and outcomes in the use cases.

Use Cases	Trust on	Blockchain Provides	Highlights
**UC1**	CA	A data-sharing platform	Presence of an existing solution stops deployment
**UC2**	Sensors	A data-sharing platform	Unclear benefits for using blockchain
**UC3**	Official documents	Transparency and trust	Clear benefits, the solution is being tested

**Table 2:** Information about the companies and their employees interviewed. Participants are denoted as **P***n*. **BC** is the abbreviation for blockchain, **mgr.** for manager, NL: Netherlands, FR: France, UK: United Kingdom, DK: Denmark.

ID	Age Range	Job Title	Level of BC Expertise (1–10)	Industrial Field	Company Size	Country
P1	31–40	Founder-R&D	7	Construction	Small	NL
P2	51–60	Supply Chain mgr.	1	Nautical	Medium	FR
P3	31–40	Business Analyst	8	Food	Small	NL
P4	51–60	Project mgr.	3	Transport/Logistics	Large	NL
P5	21–30	Vacation Job	3	Construction	Medium	NL
P6	51–60	Operations mgr.	3	Agriculture	Small	NL
P7	51–60	Director of Operations	3	Transport/Logistics	Small	NL
P8	31–40	Financial mgr.	6	Agriculture	Medium	NL
P9	31–40	General mgr.	6	Food	Small	NL
P10	41–50	CFO	4	Manufacturing	Medium	NL
P11	41–50	Supply Chain mgr.	7	Defence	Large	UK
P12	41–50	Business Analyst	9	Food	Large	NL
P13	21–30	Supply Planner	4	Manufacturing	Medium	NL
P14	21–30	Demand Planning mgr.	1	Pharmaceutical	Medium	DK
P15	31–40	Logistics Analyst	7	Agriculture	Small	NL
P16	21–30	Transportation Planner	6	Transport/Logistics	Large	NL
P17	21–30	Sourcing Specialist	3	Manufacturing	Large	NL
P18	21–30	Project mgr.	10	Software Dev.	Small	NL

**Table 3:** Reason for using the data in the companies; 18 companies in total.

Reason the Data are Used for, in the Company	Number of Companies Using the Corresponding Data	Result in %
Recording activities of process/tasks	15	83%
Informing relevant parties of the status of an activity on the supply chain	11	61%
Making decisions	9	50%
Tracking progress	1	6%
Justifying cost	1	6%

**Table 4:** Type of stakeholders in the information sharing; 18 companies in total.

Stakeholders in the Information Sharing	Number of Companies Sharing Data with the Corresponding Stakeholders	Result in %
Customers	9	50%
Other parties of the supply chain	9	50%
Only limited business partners	8	44%
Co-workers (sales/marketing/finance)	1	6%
Mostly internal	1	6%
Sales	1	6%

**Table 5:** Information sharing among stakeholders. The results highlight the portions of companies sending and receiving information to/from corresponding stakeholders.

Stakeholders in the Information Sharing	% of Company’s Information Sharing Action	
	**Sending**	**Receiving**
Manufacturers	22%	33%
Raw Material Suppliers	11%	28%
Distributors	44%	56%
Wholesalers	28%	28%
End User	33%	28%
Registrars	11%	6%
Transporters	44%	N/A
Certifiers	17%	22%
Retailers	17%	22%
Not applicable	17%	28%

**Table 6:** Companies’ current adaptation state of blockchain compared to their direct competitors.

Companies’ Current Adaptation State of Blockchain Compared to Their Direct Competitors	Portion of Companies
On a par	38.9%
Leading	27.8%
Not started/No activity	33.3%

**Table 7:** Companies’ current or future relationships with blockchain models.

Companies’ Current or Future Relationship with a Blockchain Models	Number of Companies	Result in %
Permissioned (with supply chain partners)	3	17%
Public blockchain (global, open networks)	4	22%
Private blockchain (internal to the company)	1	6%
No idea	2	14%
None are in sight	5	28%
No interest in blockchain	3	17%

## Data Availability

Data are contained within the article.

## Appendix A. Questionnaire

**Table A1:** Questionnaire used for our study (Q1–Q20). * ERP, WMS, TMS, EDI: enterprise resource system, warehouse management system, transport management system, electronic data interchange. ** API—application programming interface; usually a software interface to exchange information between separate applications or IT systems.

ID	Question
Q1	**What is the name of the company for which you work?**
Q2	**Where is your company located?**
Q3	**Would you like the answers to this form to remain anonymous?**
Q4	**What is your age group?** (21–30; 31–40; 41–50; 51–60)
Q5	**In which of the following industries does the organization you work for primarily operate?** (Construction; Nautical; Food; Transport/Logistics; Agriculture; Manufacturing; Defence; Pharmaceutical; Software development)
Q6	**Which of the following best describes your current job role/title?** (Founder-R&D; Supply Chain mgr.; Business Analyst; Project mgr.; Vacation Job; Operations mgr.; Director of Operations; Financial mgr.; CFO; General mgr.; Supply Planner; Demand Planning mgr.; Logistics Analyst; Transportation Planner; Sourcing Specialist)
Q7	**What is the amount of workers in the company you work for?**
Q8	**Which of the following roles in the supply chain best describes the organization** **for which you work?** (Producer/Manufacturer/Processor; Technology provider; Inspection; Farmer/raw material extractor; Transporter/3PL(Third-Party Logistics)/LSP(Logistic Service Providers); Fund manager & Structurer; Military; Retailer)
Q9	**Which one of the following best describes your organization’s technology stage?** (Data sharing (Sharing data with customer and/or supplier via various connector tools); Digital basic (Internal digital strategy & process improvement); Disruptions (Using digitization to transform and disrupt markets and to create a new one))
Q10	**How would you rate your company on the ability to use data and analytics to improve** **or transform the business?** (1: Not at all, 10: Data analytics expert, 0: No idea)
Q11	**In your current role, what do you use data for?**
Q12	**Which of the following best describes the interactions you have with data in your work?** (Editor (change data in the system, interacts with it); Viewer/User (visualise and use system data); Admin (in charge of the system and its good functioning))
Q13	**Which of the following technologies is the organization** **for which you work currently using? (multiple-choice)** (Clouds systems (Online storage); ERP,WMS,TMS,EDI *; API **; Connected sensors & IoT (Devices to measure and collect data); AI (Artificial intelligence); Blockchain)
Q14	**How do you process data for analysis?**
Q15	**Who do you share your information about the supply chain process with? (multiple-choice)** (Customers; Other parties of the supply chain; Only limited business partners; Co-workers-sales/marketing/finance; Mostly internal; Sales)
Q16	**How do you share information among your partners?**
Q17	**How do you store the data?**
Q18	**What is the importance of data that is stored?** (1: Nothing will happen if the data leak happens, 10: Data leak causes high damage to the company (Financial and Reputational), 0: No Idea)
Q19	**Are you using a common database (have access to the same database)** **with other parties of your supply chain?**
Q20	**Which of the following parties in the supply chain receives information from your databases** **on a regular basis (at least once a month)? (multiple-choice)** (Manufacturers; Raw Material Suppliers; Distributors; Wholesalers; End User; Registrars; Transporters; Not applicable; Certifiers; Retailers; None)

**Table A2:** Questionnaire used for our study (Q21–Q42).

ID	Question
Q21	**Which of the following parties in the supply chain sends you information** **that will impact your database? (multiple-choice)** (Standards Organizations; Retailers; Commercial; Manufacturers; Distributors; Wholesalers; Raw Material Suppliers; End User; Registrars; None; Not applicable; Certifiers)
Q22	**How many data-related mistakes were made at your company in the last 12 months?**
Q23	**How costly were those data-related mistakes (in % of your yearly turnover)?**
Q24	**How do you ensure the execution of an agreement that** **you made with other parties in the supply chain?**
Q25	**What is your level of expertise regarding blockchain? (0: Not at all, 10: Expert)**
Q26	**Have you ever developed a project around blockchain before?**
Q27	**Which of the following did you already know about blockchain can provide your business?** **(multiple-choice)** (Transparency for product or service flow; Traceable data; Connecting all parties of the supply chain within the same network; Single source of truth; Secure business agreement via Smart contracts; Security; Payments; Immutable data; Decentralized verification; Shared or equal data ownership; Inbuilt resilience and fault tolerance)
Q28	**Which of the following disadvantages of blockchaindid you already know? (multiple-choice)** (Transparency ruins business cases; Slower through consensus methods; Requires a trust problem (Trust exists; blockchain becomes inefficient); Not one size fits all (No generalization, specific solutions for each case); Distributed solution; Need active participation; Energy consumption; Centralized approach (Distributed network updated by centralized tools); Lack of understanding and implementation; Requires a cost-carrying party; Sunk Cost fallacy; Finite (Limitation on flexibility and errors))
Q29	**What do you think is the biggest opportunity for using blockchain in your company?** **(multiple-choice)** (Transparency for product or service flow; Security; Connecting all parties of supply chain within the same network; Secure business agreement via Smart contracts; Payments; Single source of truth; Immutable data; Traceable data; Decentralized verification)
Q30	**What are your organization’s barriers, if any, to greater investment** **in blockchain technology? (multiple-choice)** (Implementation/replacement of other systems; Ambiguous return on investment; Low priority for current business; Regulatory issues; Insufficient in-house knowledge or expertise; Concerns over sensitivity of competitive information; Broad adoption; The technology is new and controversial)
Q31	**Which of the following best describes how your organization currently views the relevance** **of blockchain to your organization?** (Important; Relevant; Unsure; No idea)
Q32	**Is your organization investing in exploring blockchain-based applications to** **support current or new business models now or in the future?** (Yes, now; Yes in the future <2 years; Yes in the future <5–10 years; No idea; No, not in the future)
Q33	**Which blockchain model are you focusing your activities on?** (Permissioned (with your supply chain partners); Private blockchain (internal to the company); Public blockchain (global, open networks); No idea; None are in sight; No interest in blockchain)
Q34	**Which of the following blockchain use cases is your company working on? (multiple-choice)** (Secure information transfer; Digital currency; Asset management and monitoring; Digital identification; Regulation observance; Traceability of financial flow; Digitization of data or document; Credentials and licensing; Track and trace of the product supply chain; Contracts management; None yet; No interest in blockchain; Regulation observance)
Q35	**Where is your company in its blockchain journey?** (Action (pilot under development or live already); Interest (exploring potential); Desire (preparing development); Awareness; Not sure; Not interested/compatible)
Q36	**How does your organization’s current adoption of blockchain compare to that of your** **direct competitors?** (Leading; On par; Not interested/compatible)
Q37	**What % of your yearly turnover is made available for tech investment?** (<5%; 5–10%; 10–25%; 25–50%; >50%; Unknown)
Q38	**What % of your yearly turnover is made available for blockchain investment?**
Q39	**What is your company’s current position on creating or being a participant** **of a blockchain consortium with other companies?** (Already participated in one; Considering joining one; Unsure; No interest in participating)
Q40	**What is the scale of customers or parties that demand more transparency** **in your supply chain?** (More than 50%; Between 25% and 50%; Between 10% and 25%; Between 5% and 10%; Less than 5%; No idea)
Q41	**What is your opinion about the security level of blockchain-based systems** **compared to the current system?** (Blockchain-based more secure; Don’t know; Blockchain-based less secure)
Q42	**Do you have any last comments?**

**Table A3:** Questionnaire used for our study (Q43–Q50).

ID	What is Your Level of Agreement or Disagreement with Each of the Following Statements? (0–10)
Q43	New revenue sources from blockchain and/or digital asset solutions will be seen in our sector
Q44	If my company does not implement blockchain technologies, we will miss a potential for a competitive advantage
Q45	Blockchain and/or digital assets solutions or strategies are being discussed by our business partners, suppliers, customers, and/or competitors
Q46	There exists a compelling business for utilizing blockchain technology and/or digital assets in my organization
Q47	Blockchain technology is broadly scalable and has achieved mainstream adoption
Q48	Our executive team believes blockchain and/or digital assets are overhyped
Q49	Blockchain solutions are being discussed or developed by suppliers, customers, and/or competitors to address problems in the value chain
Q50	The industry will be disrupted by blockchain technology
